# Changes in spirometric parameters after protective interventions among workers at a chlorine production plant in Iran

**DOI:** 10.4178/epih.e2020041

**Published:** 2020-06-06

**Authors:** Daryoush Pahlevan, Amir Shomali, Sara Pooryahya, Kamyar Mansori, Majid Mirmohammadkhani, Farhad Malek

**Affiliations:** 1 Social Determinants of Health Research Center, Semnan University of Medical Sciences, Semnan, Iran; 2 Department of Internal Medicine, Kowsar Hospital, Semnan University of Medical Sciences, Semnan, Iran; 3 Department of Biostatistics and Epidemiology, School of Medicine, Zanjan University of Medical Sciences, Zanjan, Iran

**Keywords:** Chlorine gas, Spirometric parameters, Protective interventions, Quasi-experimental study, Iran

## Abstract

**OBJECTIVES:**

This study was conducted to assess changes in spirometric parameters after protective interventions among workers at a chlorine production plant in Semnan, Iran during 2012-2016.

**METHODS:**

This quasi-experimental study included 100 workers at a chlorine production plant in Semnan during 2012-2016. Spirometric parameters (forced vital capacity [FVC], forced expiratory volume in 1 second [FEV1], FEV1/FVC, peak expiratory flow [PEF], and PEF occurring in the middle 50% of the patient’s exhaled volume [PEF 25-75%]) were measured in all workers before the initial intervention in 2012. Protective interventions were then implemented for 4 consecutive years and the parameters were measured annually. A multivariable linear regression model was used to assess the factors affecting spirometric parameters before and after the protective interventions in SPSS version 24.

**RESULTS:**

The mean values of all spirometric parameters significantly increased after the protective interventions (p<0.05). Multivariable linear regression showed that age (β=-0.40), body mass index (BMI) (β=0.71; 95% confidence interval [CI], 0.11 to 1.31), and type of mask (β=-7.88; 95% CI, -15.96 to -0.46) had significant effects on the mean difference in FVC. Similarly, age (β=-0.35; 95% CI, -0.70 to -0.01), BMI (β=0.80; 95% CI, 0.20 to 1.41) and type of mask (β=-8.88; 95% CI, -16.98 to -0.79) had significant associations with the mean difference in FEV1. The type of mask (β=-12.81; 95% CI, -25.01 to -0.60) had a significant effect on the mean difference in PEF.

**CONCLUSIONS:**

All spirometric parameters significantly increased in workers after protective interventions were implemented. Therefore, protective interventions to prevent respiratory disorders in workers exposed to chlorine gas are suggested.

## INTRODUCTION

Occupational health is one of the most important priorities of the World Health Organization. Occupational health focuses on various aspects of occupational health and safety, and many occupational health interventions focus on primary prevention of hazards in the workplace. However, according to a report of the International Labour Organization, nearly 2.78 million workers throughout the world die annually due to occupational injuries and work-related illnesses [1,2]. Occupational diseases are caused by exposure to chemical agents, biological agents, and physical hazards in the workplace [3]. Respiratory diseases are the most common occupational diseases and also account for about 17% of all deaths [2,4]. Chemical vapors, especially chlorine, are one of the most important risk factors for occupational respiratory diseases [5,6].

The major route of exposure to chlorine gas is inhalation. Exposure to this gas can irritate the eyes, skin, nose, throat, and mucous membranes. Eye injuries may be permanent [7]. Poisoning by chlorine can lead to simple respiratory irritation, spasm and bronchial contractions, damage to bronchioles and the walls of the air sacs (alveoli), and many other pulmonary diseases. Although there is a high chance of recovery after removal of the source of exposure to the gas and remedial action, severe and prolonged exposure can lead to permanent and irreversible damage to lung function, especially in workers in related industries [8,9]. Chronic exposure to chlorine at 15 ppm causes cough, hemoptysis, chest pain, and sore throat [10]. Various studies have suggested that chronic and prolonged exposure to chlorine is an important factor in the development of occupational asthma. Some studies have also shown that long-term exposure to chlorine causes chronic rhinitis in industrial workers, which can eventually lead to reactive airway dysfunction syndrome [11,12]. Other long-term effects of exposure to chlorine and chlorinated compounds on the respiratory system include shortness of breath, irregular breathing, irregular heartbeat, chest pain, reactive upper airway dysfunction syndrome, tooth decay, and an increased risk of colds and many other respiratory diseases [8,10].

Given the numerous complications and consequences of exposure to chlorine, designing and implementing effective protective interventions for workers in related industries seems essential. Therefore, in light of the above points and the limited studies conducted on the effects of protective interventions on lung function and spirometric parameters among workers at chlorine factories in Iran, the aim of this study was to assess the changes of spirometric parameters after protective interventions among workers exposed to chlorine gas at a chlorine production plant in Semnan, Iran during 2012-2016.

## MATERIALS AND METHODS

### Study design and participants

This quasi-experimental study was performed among 100 workers at a chlorine production plant in the city of Semnan in Iran during 2012-2016. In this study, since all workers were investigated, sampling was done in the form of a census. The inclusion criteria were working in the factory production unit throughout the study period, and having an active occupational medical record and healthy lung function at time of employment. The exclusion criteria were contraindications against spirometric testing for the worker (such as stroke or headache and chest pain in the last 6 weeks, active bloody sputum, pneumothorax, abdominal or cerebral aneurysm, recent eye surgery, abdominal or thoracic surgery, pulmonary embolism, recent cerebrovascular events), respiratory diseases (such as asthma, rhinitis, bronchitis, and emphysema), and concurrent employment at other industrial plants.

### Data collection

To collect the data, we used a checklist to gather information on variables related to demographic (age, sex, weight, height, and smoking), occupational (work experience; family history of respiratory diseases; and environmental risk factors, such as enzymes, vapors, type of mask used, and exposure to solvents and dust), and spirometric parameters including forced vital capacity (FVC), forced expiratory volume in 1 second (FEV1), peak expiratory flow (PEF), PEF occurring in the middle 50% of the patient’s exhaled volume (PEF 25-75%), and FEV1/FVC. Spirometric parameters were measured in all workers before the intervention in 2012. Then, protective interventions were implemented for 4 consecutive years and spirometric parameters were measured annually. These protective intervention measures were as follows:

(1) Electrolysis chamber insulation (overhauling the electrolysis machine, fixing small and imperceptible device leakage using a high-pressure pump, and creating exhaust ventilation in the roof of the room), (2) Fixing leakage of chlorine carrier pipes in the open area of the factory, with a shut-down of the production line and replacement of connections, (3) Emphasizing the use of filter masks by workers, and (4) Shutting down the chlorine production line when emergency repairs are performed.

We used a SpiroLab II spirometer (MIR, Rome, Italy) to measure FVC, FEV1, PEF, PEF (25-75%), and FEV1/FVC. Spirometry was performed by a trained technician under the supervision of a specialist physician based on the guidelines of the American Association for Thoracic Surgery. The mean percentage of the predicted value of each spirometric parameters was measured based on height, age, and sex. Before spirometry, the workers received a thorough explanation of the essential instructions related to the method and maneuvers, and they were asked not to smoke or eat a heavy meal for at least 1 hour before the test. For spirometry, workers were requested to stand for 5 minutes, and then special clips were placed on their noses while they were in a comfortable standing position. For each worker, 3 acceptable maneuvers were performed, and if a difference of more than 5% in the results for FVC was observed, the test was repeated up to 8 times to obtain the most accurate volume based on the predicted percent of lung function. In the present study, the values of the predicted percent of lung function was the capacity measured by spirometry divided by the anticipated capacity (according to sex, age, height, and race), and multiplied by 100. The amount of chlorine gas at workers’ stations was measured using gas-gathering sampling pumps and gas chromatography and compared with the threshold limit value.

### Statistical analysis

Data were analyzed using Stata version 14 (StataCorp., College Station, TX, USA). For descriptive analyses, the mean, standard deviation (SD), and number (%) were calculated. Then, a multivariable linear regression model was used to determine factors that affected the mean difference in spirometric parameters before and after the protective interventions. Finally, the adjusted regression coefficient (β) with a 95% confidence interval (CI) was estimated. The p-values < 0.05 were considered to indicate statistical significance.

### Ethics statement

Before data collection, the aims of the research were explained to the workers, and informed consent was then obtained. This study was performed according to the principles expressed in the Declaration of Helsinki and was approved by the Deputy of Research and Ethics Committee of Semnan University of Medical Sciences (Iran).

## RESULTS

The aim of this study was to assess changes in spirometric parameters after protective interventions among workers exposed to chlorine gas at a chlorine production plant in Semnan, Iran during 2012-2016. The total number of workers included in the study was 100. The numbers of male and female were 97 (97%) and 3 (3%), respectively. The mean values of age, weight, height, BMI, and work experience were 34.82 years, 76.60 kg, 173.54 cm, 25.40 kg/m^2^, and 7.62 years, respectively. Furthermore, 80% of workers had a family history of respiratory diseases. After the intervention programs were implemented, 65% regularly used masks. Table 1 shows the demographic and occupational characteristics of the population under study.

In 2012, spirometric parameters were measured before protective interventions were implemented, and these parameters were then measured for 4 consecutive years after the intervention. Table 2 shows the mean, SD, minimum, and maximum values of each spirometric parameters in 2012-2016. Table 3 shows the results of the paired t-test, which was used to compare the mean parameters before the interventions (2012) with the mean parameters for 4 consecutive years after the interventions (2013–2016). The mean values of all spirometric parameters (FVC, FEV1, FVC/FEV1, PEF, and PEF [25-75%]) significantly increased after the protective interventions (p<0.05).

The Kolmogorov-Smirnov test confirmed that the mean differences in each of the spirometric parameters (FVC, FEV1, FVC/FEV1, PEF, and PEF [25-75%]) before and after the protective interventions had a normal distribution (p>0.05). We then used a multivariable linear regression model to identify the factors that affected the mean differences in spirometric parameters before and after the protective interventions, because these mean differences were all continuous dependent variables with normal distributions. (Table 4). Age (β=-0.40; 95% CI, -0.75 to -0.06), BMI (β=0.71; 95% CI, 0.11 to 1.31), and type of mask (β=-7.88; 95% CI, -15.96 to -0.46) had significant effects on the mean difference in FVC. Age had a β-coefficient of -0.40 for FVC, meaning that for every 1-unit increase in the mean value of age, the FVC decreased by an average of 0.40 units. Similarly, age (β=-0.35; 95% CI, -0.70 to -0.01), BMI (β=0.80; 95% CI, 0.20 to 1.41) and type of mask (β=-8.88; 95% CI, -16.98 to -0.79) had significant associations with the mean difference in FEV1. The type of mask (β=-12.81; 95% CI, -25.01 to -0.60) also had a significant effect on the mean difference in PEF (Table 4).

## DISCUSSION

The aim of this study was to assess changes in spirometric parameters after protective interventions among workers exposed to chlorine gas at a chlorine production plant in Semnan, Iran during 2012-2016. The mean values of all spirometric parameters significantly increased after the implementation of the protective interventions (p<0.05). The multivariable linear regression model showed that age (β=-0.40), BMI (β=0.71), and type of mask (β=-7.88) had significant effects on the mean difference in FVC. Similarly, age (β=-0.35), BMI (β=0.80), and type of mask (β=-8.88) had significant associations with the mean difference in FEV1. The type of mask (β=-12.81) also had a significant effect on the mean difference in PEF.

To the best of our knowledge, this is among the first studies to investigate trends in spirometric parameters in workers exposed to chlorine gas after protective interventions, as well as associated factors, for 5 consecutive years. The implementation of protective intervention measures significantly improved spirometric parameters in workers exposed to chlorine, and these measures may provide a way to prevent permanent and irreversible damage to workers’ lung function. Due to the lack of interventional studies in this area, we were forced to compare the results with observational studies. However, in line with our results, some studies have suggested that spirometric parameters return to their normal levels after preventive measures are taken in people with respiratory disorders caused by chlorine inhalation [13-15]. Therefore, designing and implementing appropriate protective measures will slow down the process of pulmonary dysfunction and will even yield significant improvements in all spirometric parameters; hence, the importance of these protective interventions should not be neglected in workers exposed to chlorine gas in various industries.

The multivariable linear regression model showed that age, BMI, and type of mask had significant effect on mean differences in FVC and FEV1. The type of mask also had a significant effect on the mean difference in PEF. Most studies have reported negative relationships of BMI and weight with pulmonary function, such that spirometric parameters decrease as BMI or weight increases [16-18]. However, in the present study, BMI showed a significant positive association with FVC and FEV1 (p<0.05). A reason for this inconsistency may be the type of study, because most studies investigating this relationship have been cross-sectional. In this study, no statistically significant association was found between the mean values of changes in spirometric parameters, which is not consistent with some studies [19-21]. Nonetheless, the lack of statistically significant associations is consistent with the results of several other studies [22,23]. In our opinion, the most important reasons for these inconsistencies are differences in the study type and sample size. Therefore, similar interventional studies should be designed and conducted.

In our study, we was observed statistically significant negative relationships between age and the mean changes in FVC and FEV1, meaning that these parameters decreased as age increased. This finding seems reasonable. According to the results of various studies, lung capacity decreases with age as a result of age-associated declines in respiratory muscle strength; therefore, measures of lung volume and capacity such as FVC and FEV1 are affected by this process [24,25]. For this reason, particular attention should be paid to protective intervention measures in older workers.

Finally, we observed statistically significant associations between the type of masks and the mean changes in FVC, FEV1, and PEF. Specifically, these parameters showed less favorable results for participants who used inflatable and ordinary masks than for those who used filter masks (N-95). Therefore, the type of mask is an important factor that should be considered in the design and implementation of protective intervention programs.

The study has several strengths and limitations. To the best of our knowledge, the present study is the first interventional study to investigate the effects of protective interventions on lung function among workers exposed to chlorine gas in an industrial setting in Iran. The second strength of this study is the follow-up of workers’ lung function and spirometric parameters for 4 consecutive years after the initial interventions. Nonetheless, this study, like many others, has several limitations that should be considered when interpreting the results. The most important limitation of this study is the lack of a control group with random allocation for comparison because this was a quasi-experimental study. Secondly, the workers who participated in this study may be exposed to chlorine gas outside the factory in different ways, and this issue was not considered in our study.

In conclusion, this study showed that all the spirometric parameters (FVC, FEV1, FVC/FEV1, PEF, and PEF [25-75%]) significantly increased in workers after protective interventions were implemented. Furthermore, age, BMI, and the type of mask were the most important factors affecting spirometric parameters. Therefore, it is suggested that protective interventions should be implemented in a way that takes the above factors into account to prevent functional respiratory disorders in workers exposed to chlorine gas.

## Figures and Tables

**Table 1.** Demographic and occupational characteristics of the workers in this study (n=100)

Characteristics	n (%) or mean±SD (max-min)
Demographic factor	
Age (yr)	34.82±7.44 (65-25)
Weight (kg)	76.60±12.55 (51-105)
Height (cm)	173.54±7.42 (192-152)
Body mass index (kg/m^2^)	25.40±3.68 (35.63-16.65)
Sex	
Female	3 (3.0)
Male	97 (97.0)
Smoking	
Yes	12 (12.0)
No	88 (88.0)
Occupational factor	
Family history of respiratory disease	
Yes	17 (17.0)
No	83 (83.0)
Work experience (yr)	7.62±4.41 (25-1)
Exposure to environmental risk factors (enzymes, vapors, solvents, dust)	
Yes	80 (80.0)
No	20 (20.0)
Regular use of masks	
Yes	65 (65.0)
No	35 (35.0)
Type of mask	
Filtered mask (N-95)	12 (12.0)
Inflatable mask	18 (18.0)
Ordinary mask	70 (70.0)

SD, standard deviation; Max, maximum; Min, minimum.

**Table 2.** Values of spirometric parameters by year, 2012-2016

Parameters (L/S)	Intervention	Year	n	Mean±SD	Min	Max
FVC	Before	2012	100	85.86±6.22	70	98
	After	2013	100	100.99±11.35	64	132
	After	2014	100	99.74±1.57	72	140
	After	2015	100	88.57±11.24	66	116
	After	2016	100	96.75±16.26	67	182
FEV1	Before	2012	100	80.51±4.38	68	89
	After	2013	100	98.06±11.84	63	127
	After	2014	100	97.65±12.59	69	143
	After	2015	100	89.16±11.52	64	129
	After	2016	100	96.24±16.15	60	182
FEV1/FVC	Before	2012	100	72.19±4.83	63	87
	After	2013	100	81.86±6.38	65	95
	After	2014	100	82.65±6.72	62	95
	After	2015	100	84.19±6.63	63	96
	After	2016	100	84.43±7.48	66	115
PEF	Before	2012	100	82.73±9.49	60	105
	After	2013	100	103.77±16.15	67	143
	After	2014	100	103.68±17.73	57	168
	After	2015	100	98.77±16.04	68	146
	After	2016	100	108.30±23.12	63	199
PEF (25-75%)	Before	2012	100	62.98±9.52	46	85
	After	2013	100	86.53±22.28	44	157
	After	2014	100	85.97±27.42	45	172
	After	2015	100	86.98±24.96	39	159
	After	2016	100	93.74±27.41	36	186

L/S, liter per second; SD, standard deviation; Max, maximum; Min, minimum; FVC, forced vital capacity; FEV1, forced expiratory volume in 1 second; PEF, peak expiratory flow; PEF (25-75%), PEF occurring in the middle 50% of the patient’s exhaled volume.

**Table 3.** Changes of spirometric parameters after the intervention

Variables (L/S)	n	Before intervention (2012)	After intervention (mean 2013-2016)	Mean change (after-before)	p-value^1^
FVC	100	85.86±6.22	96.51±9.84	10.65	<0.001
FEV1	100	80.51±4.38	95.27±10.64	14.76	<0.001
FEV1/FVC	100	72.19±4.84	83.28±5.92	11.10	<0.001
PEF	100	82.73±9.49	103.63±15.56	20.90	<0.001
PEF (25-75%)	100	62.98±9.52	88.30±20.21	25.32	<0.001

Values are presented as mean±standard deviation. L/S, liter per second; FVC, forced vital capacity; FEV1, forced expiratory volume in 1 second; PEF, peak expiratory flow; PEF (25-75%), PEF occurring in the middle 50% of the patient’s exhaled volume.

1 Paired t-test.

**Table 4.** Factors affecting the mean changes in spirometric parameters after the interventions by a multivariable linear regression model

Variables	FVC	p-value	FEV1	p-value	FEV1/FVC	p-value	PEF	p-value	PEF (25-75%)	p-value
Age	-0.40 (-0.75, -0.06)	0.022	-0.35 (-0.70, -0.01)	0.043	-0.03 (-0.28, 0.21)	0.765	-0.17 (-0.74, 0.39)	0.549	-0.34 (-1.08, 0.40)	0.367
Body mass index	0.71 (0.11, 1.31)	0.022	0.80 (0.20, 1.41)	0.010	0.16 (-0.27, 0.59)	0.459	0.50 (-0.50, 1.50)	0.325	0.91 (-0.40, 2.21)	0.170
Work experience	0.11 (-0.47, 0.69)	0.716	-0.01 (-0.60, 0.57)	0.962	-0.06 (-0.48, 36.00)	0.782	-0.35 (-1.32, 0.61)	0.472	0.06 (-1.20, 1.32)	0.926
Smoking	5.60 (-1.90, 13.11)	0.141	6.50 (-1.02, 14.03)	0.089	1.50 (-3.90, 6.89)	0.583	10.72 (-1.72, 23.16)	0.090	11.03 (-5.22, 27.30)	0.181
Family history of respiratory disease	1.14 (-4.66, 6.96)	0.698	-2.25 (-8.08, 3.58)	0.455	-3.77 (-7.96, 0.40)	0.076	-6.74 (-16.39, 2.91)	0.169	-8.19 (-20.80, 4.41)	0.200
Exposure to environmental risk factors	2.23 (-3.27, 7.74)	0.423	1.85 (-3.67, 7.37)	0.507	0.71 (-3.24, 4.67)	0.721	-5.61 (-14.74, 3.52)	0.226	0.45 (-11.48, 12.39)	0.940
Regular use of masks	1.32 (-3.65, 6.30)	0.599	0.97 (-4.01, 5.97)	0.699	-0.05 (-3.63, 3.52)	0.977	-4.39 (-12.65, 3.86)	0.294	0.41 (-10.37, 11.21)	0.939
Filtered mask (N-95)	1.00 (reference)	-	1.00 (reference)	-	1.00 (reference)	-	1.00 (reference)	-	1.00 (reference)	-
Inflatable mask	-7.88 (-15.96, -0.46)	0.044	-8.88 (-16.98, -0.79)	0.032	-0.19 (-6.01, 5.61)	0.948	-8.94 (-22.33, 4.44)	0.188	-7.70 (-25.20, 9.80)	0.385
Ordinary mask	-6.41 (-13.78, 0.95)	0.087	-6.33 (-13.71, 1.05)	0.092	-.011 (-5.40, 5.18)	0.968	-12.81 (-25.01, -0.60)	0.040	-5.57 (-21.52, 10.38)	0.489

Values are presented as β-coefficient (95% confidence interval). FVC, forced vital capacity; FEV1, forced expiratory volume in 1 second; PEF, peak expiratory flow; PEF (25-75%), occurring in the middle 50% of the patient’s e haled volume; CI, confidence interval.
